# Hypoglycemic Effects of Oat Oligopeptides in High-Calorie Diet/STZ-Induced Diabetic Rats

**DOI:** 10.3390/molecules24030558

**Published:** 2019-02-03

**Authors:** Jun-bo Wang, Xin-ran Liu, Si-qi Liu, Rui-xue Mao, Chao Hou, Na Zhu, Rui Liu, Hui-juan Ma, Yong Li

**Affiliations:** Department of Nutrition and Food Hygiene, School of Public Health, Peking University, Beijing 100191, China; bmuwjbxy@bjmu.edu.cn (J.-b.W.); liuhappy07@163.com (X.-r.L.); lsqdeyouxiang@126.com (S.-q.L.); maoruixue@163.com (R.-x.M.); houchao_HC@163.com (C.H.); summer920503@163.com (N.Z.); liuruipku@163.com (R.L.); mhjmhjmhj717@163.com (H.-j.M.)

**Keywords:** oat oligopeptides, hypoglycemic effect, oral glucose tolerance test

## Abstract

The study was aimed to determine whether treatment with oat oligopeptides (OOPs) could modulate hyperglycemia related to type 2 diabetes mellitus (T2DM) in Sprague–Dawley (SD) rats. Diabetic SD rats modeling by a joint effect of high-calorie diet for 45 days and twice intraperitoneal injection of 30 mg/kg streptozotocin at one-week interval were observed with or without OOPs administration (0.25, 0.50, 1.00, and 2.00 g/kg Body Weight) for 12 weeks. Fasting blood glucose (FBG), oral glucose test tolerance (OGTT), serum insulin, level of antioxidant, and hepatic enzymes were measured. In addition, frequency of micturition was recorded in this study for the first time. It was observed that the administration of OOPs (2.00 g/kg Body Weight) resulted in a significant decrease (*p* < 0.05) in FBG since 6th week and a significant decrease (*p* < 0.05) in the OGTT-AUC on 6th and 10th week. In addition, the administration of OOPs (2.00 g/kg Body Weight) reduced HOMA-IR index and 24-h urine volume significantly (*p* < 0.05) whereas increased SOD activity significantly (*p* < 0.05). These results suggested that OOPs may have a hypoglycemic effect in diabetic rats.

## 1. Introduction

Diabetes mellitus (DM) is a group of endocrine and metabolic disorders with booming prevalence worldwide, caused by insufficient insulin secretion or decreased sensitivity to insulin in peripheral tissues. According to the data of International Diabetes Federation (IDF) [1], about 425 million adults in the world have diabetes in 2017, and the diabetic population is expected to reach 693 million people by 2045. In addition, the prevalence of impaired glucose tolerance (IGT) among adults aged 20–79 in 2017 was 7.3%, and it is expected to reach 8.3% by 2045. As one of the leading causes of death in the world, about four million people (20–79 years old) died of diabetes and its complications in 2017, accounting for 10.7% of the total worldwide deaths. Diabetic patients suffering from various symptom, such as increased urine output, thirst, weight loss, and weakness can’t have a normal quality of life like others. On the other hand, in 2017, the healthcare expenditure of diabetes and its complications around the world was hundreds of billions of dollars. It can be seen that diabetes not only seriously endangers human health, but also brings heavy financial burden to individuals, families and society. Hypoglycemia and prevention of diabetes and complications have become the focus of scientific research.

In many factors that influence the development of diabetes, nutritional treatments, compared with factors such as genetic, can be modified and proved effective [2,3]. Many studies have shown that oats, as a recommended whole grains, have remarkable hypoglycemic effects [4,5,6,7]. In 2014, Bao and others came to a conclusion by 15 randomized controlled studies (from the US, Canada and Europe) that compared with foods such as wheat, daily intake of more than 3 mg oat β-glucan (equivalent to more than 60 g oats) for more than eight weeks can significantly reduce fasting insulin, blood glucose and glycosylated hemoglobin levels [8]. A meta-analysis of randomized controlled trials has found that oat intake resulted in a greater decrease in fasting glucose and insulin of subjects (*p* < 0.05) than control group, but β-glucan extract intake did not [9]. This shows that other components in oats may play an important role in blood glucose regulation.

In recent years, it has been found that bioactive peptides have many remarkable physiological functions and activities [10]. Compared with proteins, bioactive peptides have simpler structures, higher stability, and lower or no immunogenicity. Domestic and foreign scholars have isolated numerous peptides with hypoglycemic function from natural plants and animals [11]. The protein content in oats is 10–20% [12,13], which is about twice that of rice, wheat and cornmeal. Oat protein contains 18 kinds of amino acids, and the essential amino acid composition is relatively balanced and comprehensive. For example, lysine is as high as 0.75 g/100 g, which is inferior to other grain crops [14]. Therefore, oat protein is considered to be a better grain protein [15], and also a raw material for preparing high-quality OOPs. At present, however, there is little research on the effect of oat oligopeptides on blood glucose. Therefore, the main purpose of this study is to explore the hypoglycemic effect of OOPs on diabetes mellitus. Meanwhile, there is no study on micturition frequency measurement in diabetic rats as yet. This study will also focus on micturition frequency changes in diabetic rats with OOPs intervention.

## 2. Results

### 2.1. Effect of OOPs on General State and Body Weight

**Figure 1:**
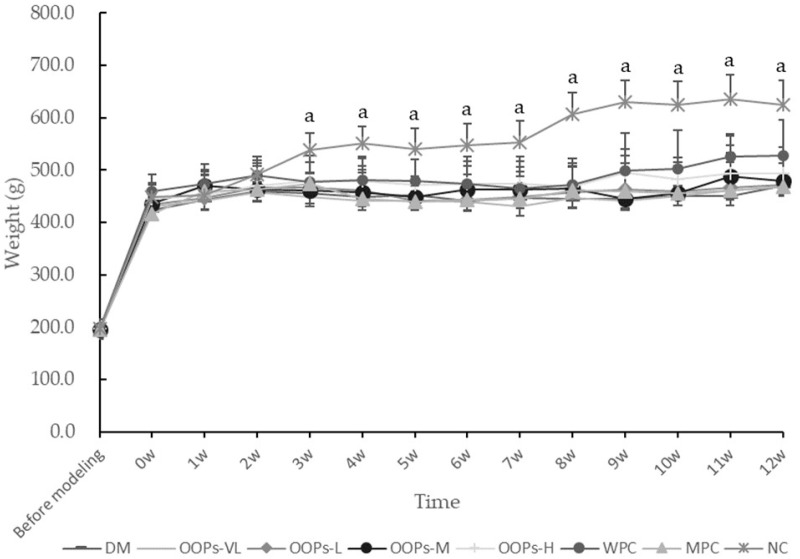
Effect of OOPs on body weight.

**Figure 2:**
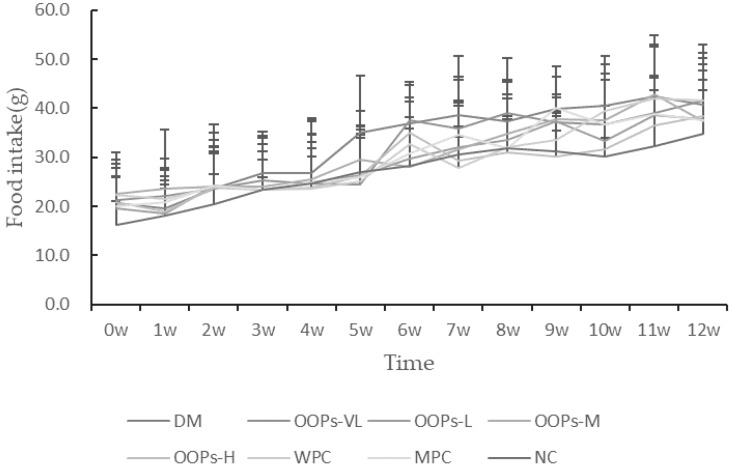
Effect of OOPs on food intake.

Normal rats have smooth hair, shiny luster, active and fecal columnar formation, while diabetic rats show a weak state, rough and dull hair, unresponsiveness, decreased activity and diarrhea in individual rats. The body weight of each group was as shown in Figure 1. There were no significant differences in body weight among the groups before modeling. From the 3th week, the body weight of each diabetic model group was lower than that of the normal group significantly, indicating that the regular growth of rats in diabetic model groups was inhibited. Although there were no differences in body weight among diabetic rats groups, the mental status and defecation of OOPs intervention rats were better than the diabetic model control group. As shown in Figure 2, food intake made no diference.

All the values are presented as mean ± SD (*n* = 8). The data were analyzed for significance of differences by one-way analysis of variance test. ^a^ *p* < 0.05 versus MC rats. (OOPs-VL, OOPs-L, OOPs-M and OOPs-H refer to that rats were treated OOPs at 0.25, 0.50, 1.00, and 2.00 g/kg Body Weight for 12 weeks respectively). The effects of OOPs on urine pH are presented in Table 1. A significant effect of OOPs-H on body weight and plasma parameters of uric acid and albumin was found (all *p* < 0.0001) that was higher in obese compared with lean Zucker rats,

### 2.2. Effect of OOPs on FBG

There were no significant differences in FBG levels among all groups before modeling. All of them were in the normal range (5.4 ± 0.36 mmol/L). Then the FBG levels of the diabetic rats were significantly higher than those in the normal control group (*p* < 0.05) as the diabetic rats model has been formed (shown in Table 2).

With the continuous intervention, the FBG levels in OOPs treatment groups showed a decreasing trend compared with the MC group. Notably, OOPs-H group had a significant decrease of FBG compared with the MC group since 6th week (*p* < 0.05). The FBG in all OOPs treatment groups had a significant decrease on 8th week, while OOPs-VL group also showed the hypoglycemic effect on the 6th week (*p* < 0.05). Also, it can be seen that some groups show significantly effects of lowering FBG compared with WPC or MPC which will be mentioned later in the Discussion section.

### 2.3. Effect of OOPs on OGTT-AUC 

The OGTT was conducted at 6th week and 10th week and OGTT-AUC was calculated based on blood glucose of 0 h, 0.5 h, 1 h, and 2 h after oral glucose administration in rats (Table 3). OGTT-AUC of diabetic groups was significantly higher than NC group. Remarkably, OGTT-AUC of OOPs-H group were significantly lower than that in MC group at 6th week and 10th week. Table 4 shows that the OGTT-2nd hour glucose level of the MC group was significantly higher than that in NC group (*p* < 0.05). Relative to the MC group, the OGTT-2nd hour glucose of OOPs-H group were significantly reduced (*p* < 0.05 for both 6th week and 10th week).

### 2.4. Effect of OOPs on Insulin Metabolism

**Figure 3:**
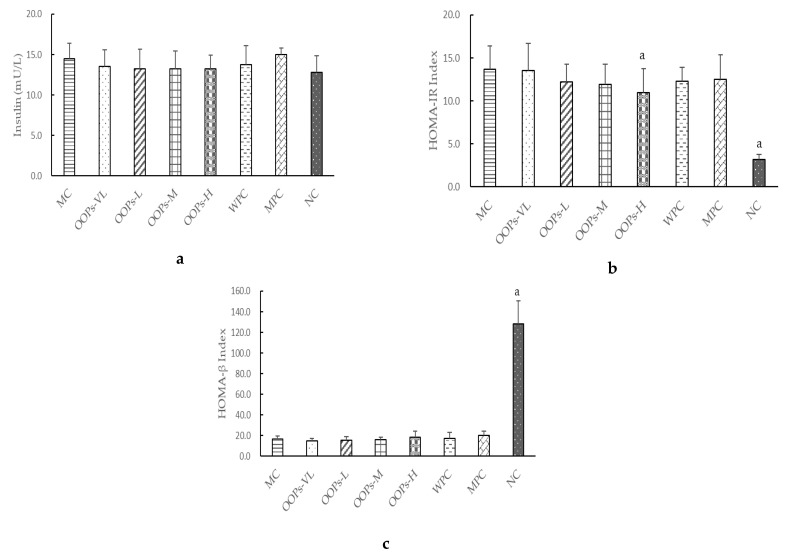
Effect of OOPs treatment on the fasting insulin (**a**), HOMA-IR-Index (**b**), and HOMA-β Index (**c**). Values are expressed as the means ± SD of 8 rats in each group. ^a^ *p* < 0.05 versus MC group.

As shown in Figure 3a, there was no significant difference in serum insulin between the groups. Homoeostasis model assessment-insulin resistance (HOMA-IR) is an index used to evaluate the level of insulin resistance. It can be seen that HOMA-IR Index of OOPs-H group was significantly lower than that of MC group (Figure 3b).

Homoeostasis model assessment beta-cell function (HOMA-β) is an indicator for evaluating the function of islet beta cells. It can be seen in Figure 3c that the islet beta cells’ function was damaged in the diabetic rats, but there was no significant difference among the OOPs intervention groups and the model control group.

### 2.5. Effect of OOPs on SOD and MDA

The superoxide dismutase (SOD) activity of model control group was lower than that of NC group, indicating that the antioxidant capacity of diabetic rats decreased, while the SOD values of four OOPs treatment groups were higher than that of model control group. The SOD activity of OOPs-H group was significantly higher than that of MC group (*p* < 0.05), indicating that OOPs intervention could improve the antioxidant capacity of diabetic rats. There was no significant difference in the level of malondialdehyde (MDA) among all groups (Table 5).

### 2.6. Effect of OOPs on Liver and Kidney Function

**Figure 4:**
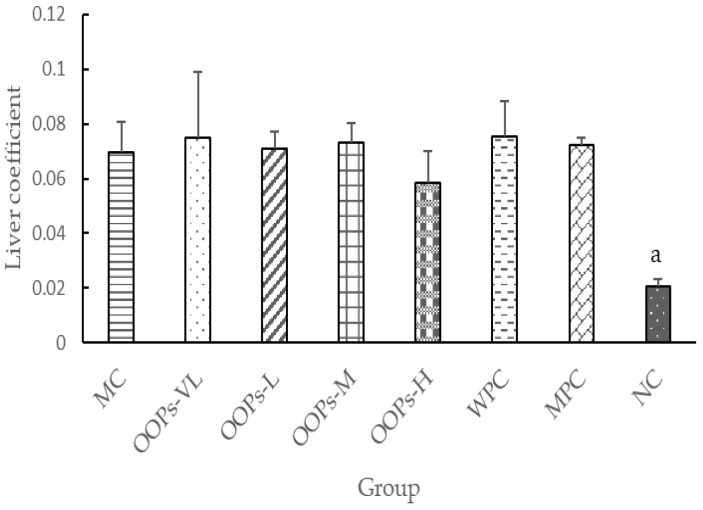
Effect of OOPs treatment on the liver coefficient. Values are expressed as the means ± SD of eight rats in each group. ^a^ *p* < 0.05 versus MC group.

Liver coefficient is the ratio of weight of the liver (g) to body weight (g) reflecting to the relative volume of the liver. The liver coefficient in all diabetic groups was significantly higher than that of the NC group, which means liver function of diabetic rats was impaired (Figure 4). But there is no significant difference of liver coefficient among these diabetic groups.

Compared with the normal control group, the serum lanine aminotransferase (ALT) and aspartate aminotransferase (AST) levels of the diabetic groups increased to different degrees, but there is no significantly difference among diabetic groups. Interestingly, the ALT and AST were both higher in MPC group than others while there is no significant difference in statistics. (Table 6).

In Table 7 it can be seen that both the micturition frequency and urine volume in diabetic groups was significantly higher than normal control group. Relative to the MC group, the urine volume of OOPs-H group was significantly reduced. 

However, there were no significant differences in micturition frequency among the OOPs treatment groups when compared with MC group, it can be seen a slight downward trend in OOPs treatment groups. 

**Figure 5:**
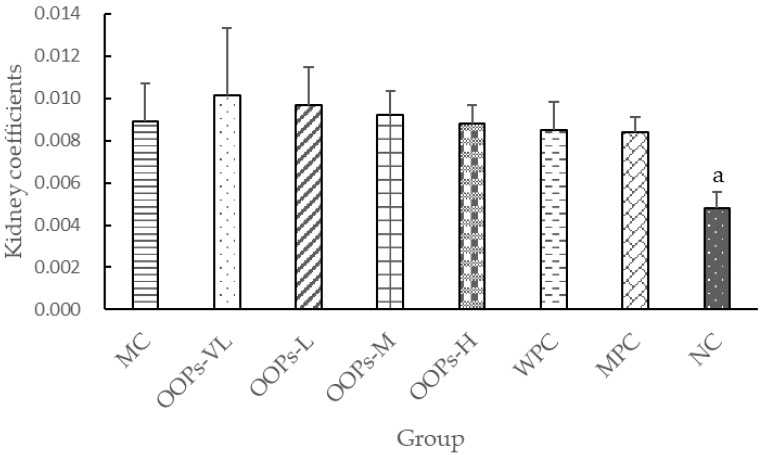
Effect of OOPs treatment on the kidney coefficient. Values are expressed as the means ± SD of 8 rats in each group. ^a^ *p* < 0.05 versus MC group.

As shown in Figure 5, the kidney coefficients of the diabetic groups were significantly higher than that in the NC group, but there was no statistical difference among diabetic groups.

## 3. Discussion

Diabetes is a complex and refractory disease with high morbidity, high disability and high mortality which can cause many complications such as diabetic ophthalmopathy, diabetic nephropathy and diabetic foot. Past studies have found oats and many bioactive peptides with obvious antidiabetic effects. The oat oligopeptides used in this study is mainly composed of small peptides of 3–4 amino acids. Theoretically, it is better to oat and oat peptides in physiological function and physicochemical properties, so the effects of OOPs on hyperglycemia, insulin metabolism, oxidative stress and hepatic enzymes were evaluated for the first time. Beyond that, micturition frequency as a criterion of measuring the life of quality of diabetic animal were evaluated firstly. 

The method of diabetic animal model construction is very critical. Alloxan, used to induce the diabetic model, was also found to cause liver and kidney injury, followed by animals’ death caused by ketosis. A better diabetic animal model similar to the adult patient of the general type 2 diabetes can be establishing by long-term high-calorie or high-fat chow combined with low-dose STZ [16,17,18]. The main mechanism causing the experimental diabetes is the induction of insulin resistance through the consumption of the high calorie diet, followed by selective damage to the pancreatic β cells of animals caused by STZ. In this study, diabetic SD rats were modeled by the joint effect of high-calorie diet administration for 45 days and twice intraperitoneal injection of 30 mg/kg streptozotocin at a one-week interval, resulting in a long-term and stable state of hyperglycemia. Throughout the study, compared with the NC group rats, the diabetic rats exhibited significantly higher FBG levels, poorer OGTT-AUC, more serious insulin resistance, more severe liver impairment and other typical characteristics of type 2 diabetes mellitus.

OOPs treatment can reduce FBG significantly, as shown by a 12.38–14.77%% decrease in the FBG in OOPs-H group (*p* < 0.05) compared with the MC group. Although the hypoglycemic effect was not shown in all the OOPs groups at all timepoints, the results still suggested OOPs can reduce FBG even beyond that of metformin treatment at the 6th week. Compared with the WPC group, the OOPs-H group exhibited significantly lower FBG at the 6th week, 8th week and 12th week. This implies the hypoglycemic effect of OOPs-H was not due to the extra protein supplementation but the intrinsic activity of the peptides in oats. As for the MPC control group, a significantly reduced FBG only occurred at the 8th week. This might because of a relatively insufficient dosage of metformin (200 mg/kg body weight) that was not enough to correct such a stubborn hyperglycemia state. That also demonstrated the hypoglycemic effect of OOPs is notable. Previous studies have shown that glucose tolerance can be used as an important indicator for screening and diagnosing diabetes and complications [19,20]. In our study, OOPs treatment can reduce OGTT-AUC which reflects glucose tolerance in diabetic rats. Meanwhile, the WPC group also had a significantly reduced OGTT-AUC at the 6th week compared with the MC group, reminding us that protein supplementation may have an effect on glucose tolerance and the mechanism of improving glucose tolerance in OOPs-H group may partly because of this. Postprandial hyperglycemia is a risk factor for vascular complications in type 2 diabetes and can significantly aggravate fasting hyperglycemia in diabetic patients [21,22]. In our study, we used the 2nd hour glucose in OGTT test to reflect the postprandial glucose in diabetic patients. The 2nd hour glucose concentration increased to more than 20 (mmol/L) in diabetic rats, but not in the OOPs-H group, whose glucose levels were significantly lower than those in the MC group. Therefore, FBG, OGTT-2nd hour glucose and OGTT-AUC are important indicators in diabetes control, and OOPs treatment has made some beneficial effects in all the above mentioned aspects.

As we all know, one of the main characteristics of type 2 diabetes is insulin resistance. The study found that the effect of OOPs intervention on serum insulin in diabetic rats was not obvious, but the HOMA-IR index representing the insulin resistance level was significantly reduced. That means the mechanism of OOPs hypoglycemia was not through promoting insulin secretion, but rather by changing the insulin resistance level thus leading to increased uptake and utilization of glucose.

The exertion of insulin’s function is influenced by many factors. The change of oxidative stress is one of the important reasons for the decline of islet cell function, the decrease of insulin sensitivity and insulin resistance. That antioxidants can improve insulin resistance has been repeatedly proven [23]. A study found that oat protein can increase the activity of GSH-Px, SOD and CAT, increase glutathione synthesis, and reduce the production of reactive oxygen species [24]. It is suggested that oat oligopeptides may regulate blood sugar and prevent complications through improving the level of oxidative stress. In the study, SOD activity has been significantly elevated, which has proven the mechanism of action is related to oxidative stress.

In addition, elevations of ALT in MPC group remind us that long-term use of metformin must be monitored, especially in those diabetic patients with liver diseases though the elevations show no significant difference with the NC group in this study. More exploration of this issue is needed. 

People with diabetes often suffer from polyuria and frequent urination. Due to the increase in blood glucose, the level of primary urine glucose is so high that the glomerulus isn’t capable of reabsorbing all the glucose into the blood, therefore, the osmotic pressure in the urine is increased, and the water is naturally infiltrated, resulting in osmotic diuresis in diabetic patients. In our study, a significant decrease in urine volume in OOPs-H group reflects a good control of blood glucose. Lowered urine pH in diabetic rats may result from the increased secretion of H+ from the renal tubules, while is the activation of the Na+ − H+ exchanger (NHE) in the renal proximal tubules and is closely linked to the renovascular damage [25]. Thus, remarkably increased levels of urine pH in diabetic rats with OOPs-H intervention are expected to have had a positive effect on renal function. Meanwhile, we found that OOPs treatment can reduce the micturition frequency to some degree and reduce the urine volume significantly. That is a novel indicator which is meaningful for improving the quality of life of type 2 diabetic patients. 

Concluding the testing results, in addition to the mechanisms already described before which may improve antioxidant activity and reduce insulin resistance, the amino acid composition and sequence may also affect the activity of peptides. A study has shown that biological function of oat protein may be due to its lower lysine/arginine ratio and lysine/glycine ratio [26]. Besides, studies have found that different amino acid components can affect the glucose-lowering effect [27]. The two amino acids with the highest content in OOPs were glutamic acid (13.607 g/100 g) and aspartic acid (5.623 g/100 g), indicating that the hypoglycemic activity of OOPs may be related to the high concentrations of glutamic acid and aspartic acid. This research is the first study on oat oligopeptide, and the underlying mechanism needs further investigation. The limitations of this study are a lack of in-depth exploration of possible kidney-related mechanisms and a more appropriate dose of metformin should be determined.

## 4. Materials and Methods

### 4.1. Materials and Reagents

Oat oligopeptides is a mixture of small bioactive peptides obtained through enzymolysis of oat bran. It came from the Weoat Group AG (Inner Mongolia, China). The amino acid composition was analyzed by an automatic amino acid analyzer (H835-50, Hitachi, Tokyo, Japan). Identification of OOPs were showed that amino acids accounted for 67 wt% of the total peptides. The amino acid composition is shown in Table 8. In addition, 92.05% of the OOPs sample had a molecular weight of less than 1000 Dalton. 

The doses of OOPs in this study were determined based on a randomized clinical trial taking into account the peptides content in oats and the dose conversion relationship between humans and rats [28].

Assay kits used for the determination of blood glucose, serum insulin, ALT and AST were purchased from Yingkexinchuang Science and Technology Ltd. (Macau, China). The SOD and MDA detection kits were purchased from Nanjing Jiancheng Biotechnology Institute (Nanjing, China). 

Streptozotocin (99.8% purity) was purchased from Sigma Chemical Co. (St Louis, MO, USA). Glucose (99.8% purity) was purchased from Beijing Jinhui Taiya Chemical Reagent Co., Ltd. (Beijing, China). Metformin hydrochloride extended-release tablets were purchased from Sino-American Shanghai Squibb Pharmaceuticals Ltd. (Shanghai, China, national medicine permit number: H20023370). Whey Protein Lacprodan 80 (79.2% purity) was purchased from Beijing Zhongbai Venture Chemical Products Co., Ltd. (Beijing, China). All other reagents used in this study were of analytical grade. High-calorie feed (52.6% basic feed, 10% lard, 15% sucrose, 15% yolk powder, 5% casein, 1.2% cholesterol, 0.2% sodium cholate, 0.6% calcium bicarbonate and 0.4% stone powder) were processed by Beijing Keao Xieli Co. Ltd. (Beijing, China) (feed certificate number: 11002900030558) according to the evaluation method of auxiliary hypoglycemic function of health food (National Food and Drug Administration [2012]107). Basic feed was processed by Beijing Keao Xieli Co. Ltd. (Beijing, China) according to the national standard (GB 14924.3-2010).

### 4.2. Animals and Feeding Conditions

Male Sprague-Dawley rats (200 ± 10 g) were obtained from the Animal Service of Health Science Center (Peking University, Beijing, China) and they were housed two per cages with free access to chow in a Specific Pathogen Free (SPF) room. The production certificate number and use license number of the rats are SCXK (Beijing) 2016–0010 and SYXK (Beijing) 2016–0041, respectively. The SPF conditions involved controlled temperature (25 ± 1 °C), relative air humidity (50–60%) and 12-h light/dark cycles. All animal procedures were handled in accordance with Regulations on the Management of Experimental Animals in Beijing and approved by the Ethics Committee of Peking University (ethics No.: LA2017189).

### 4.3. Establishment of a Type 2 Diabetic Rat Model

Healthy adult SD male rats were adapted to diet and environment for one week before the experiments began. Then they received intraperitoneal injections of low-dose STZ (30 mg/kg) twice after 45-days on a high-calorie diet. The interval between the two injections was one week. Three days after the last injection, tail vein blood glucose was measured after fasting for 5 h. Rats having blood glucose between 10–25 mmol/L were considered as diabetic rats were used for our study.

### 4.4. Experimental Design

**Figure 6:**
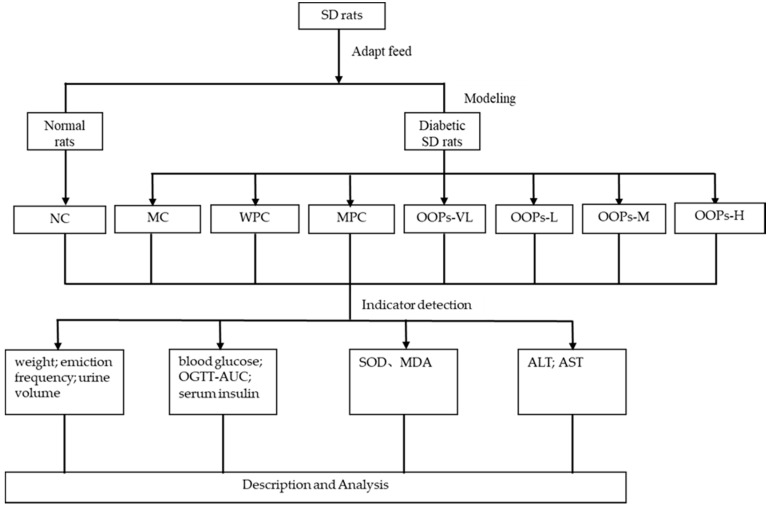
Schematic diagram of the study protocol.

Sixty-four rats were divided into two major groups which included a normal rats group (NC, *n* = 8) and a diabetic rats group (*n* = 56). Then, according to the baseline plasma glucose, the diabetic rats group was further randomly subdivided into seven groups (*n* = 8), the model control group (MC), the whey protein group (WPC), the metformin positive control group(MPC), and four OOPs treatment groups (including very low-dose group (OOPs-VL), low-dose group (OOPs-L), medium-dose group (OOPs-M) and high-dose group (OOPs-H)). Metformin here was used as a reference hypoglycemic drug, and whey protein was used as a reference protein at a dose of 1.0 g/kg Body Weight. The metformin positive control group were administrated with metformin at an initial dose of 50 mg/kg body weight and increased by 50 mg/kg body weight every two weeks, and the maximum dose is 200 mg/kg body weight. The four OOPs treatment groups were administered with OOPs at a dose of 0.25, 0.50, 1.00 and 2.00 g/kg Body Weight respectively, whereas the normal control group were received distilled water. All rats were received corresponding treatment by gavage once a day for 12 weeks with the 10 mL/kg body weight volume. Animals were free to eat and drink during the experiment. A schematic diagram of the study protocol in shown in Figure 6.

### 4.5. Observation of General State

The state of each group of rats including coat color, mental state and daily activities, was observed daily, and the food intake, water intake and body weight were regularly recorded. After the end of the experiment, main organ weights were weighed to calculate the organ coefficient.

### 4.6. Record of Micturition Frequency and Urine Volume

Micturition frequency: Six rats in each of the NC group, DM group, MPC group and four OOPs group were randomly selected to record the micturition frequency within 24 h by using a metabolic cage. Every rat had a separate cage with a funnel below to collect the urine. With a thin paper towel laid under the cage, we can record the timing of micturition immediately by observing whether the paper towel gets wet. Once the mouse urinates, we immediately record and replace a new paper towel for the next observation.

Urine volume: Six rats in each of the NC group, DM group, MPC group and four OOPs group were randomly selected to record the urine volume within 24 h by using a metabolic cage. Every rat had a separate cage with a funnel below to collect the urine. With a collection tube under the cage, we can record the urine volume by using a measuring cylinder.

### 4.7. Determination of Fasting Plasma Glucose Levels and Oral Glucose Tolerance Test

After fasting for 5 h, the FBG in the tail vein were measured at the 0th, the 4th, the 6th, the 8th, the 10th and the 12th week. Oral glucose tolerance tests were conducted in the 6th and 10th week. After fasting for 12 h, the rats were gavaged with 50% glucose solution at a dose of 2.00 g/kg Body Weight. Simultaneously, blood glucose levels were measured after 0 h, 0.5 h, 1 h and 2 h. The area under the blood glucose curve was calculated using the formula below:

OGTT-area under curve (OGTT-AUC) = 0.25 × (0 h FBG (mmol/L) + 2 × 0.5 h FBG (mmol/L) + 3 × 1 h FBG (mmol/L) + 2 × 2 h FBG (mmol/L)).(1)

HOMA-IR and HOMA-β were calculated by the HOMA method using the following formula:

HOMA-β = (20 × FINS (µIU/mL))/(FBG (mmol/L) − 3.5). HOMA-IR = FBG (mmol/L) × FINS(µIU/mL)/22.5(2)

### 4.8. Biochemical Analysis

The levels of ALT, AST in serum were detected using an AU480 automatic biochemistry analyzer (Olympus, Tokyo, Japan).

### 4.9. Statistical Analysis

Data were expressed as means ± SD ($\bar{x}$ ± s). Statistical comparisons were performed with the SPSS 24.0 software (IBM Corp, Armonk, NY, USA) using one-way analysis of variance (ANOVA). A value of *p* < 0.05 was considered statistically significant.

## 5. Conclusions

This study demonstrate that OOPs treatment can significantly reduce FBG and OGTT-AUC in diabetic rats induced by high-calorie and low dose of STZ. OOPs treatment to significantly improve insulin resistance. Similarly, oxidative stress was improved in the OOPs treatment group. It is indicated the effect of OOPs treatment on T2DM can be potentially mediated by an inhibition of oxidative stress and improved insulin resistance. We also invented a method to measure the micturition frequency in diabetic rats. Our findings indicate a beneficial effect of OOPs in glucose metabolism, which may aid in the design of a new therapies for T2DM in humans.

## Figures and Tables

**Table 1:** Effect of OOPs treatment on the urine pH.

Group	Urine pH
MC	6.2 ± 0.3
OOPs-VL	6.2 ± 0.3
OOPs-L	6.4 ± 0.5
OOPs-M	6.6 ± 0.6
OOPs-H	6.8 ± 0.6 ^a^
WPC	6.3 ± 0.6
MPC	6.5 ± 0.7
NC	7.0 ± 0.5 ^a^

Values are expressed as the means ± SD of 8 rats in each group. ^a^ *p* < 0.05 versus MC group.

**Table 2:** Effect of OOPs intervention on fasting blood glucose in rats.

Group	Fasting Blood Glucose (mmol/L)					
	0th Week	4th Week	6th Week	8th Week	10th Week	12th Week
MC	19.49 ± 3.48	18.67 ± 1.33	19.62 ± 1.60	21.42 ± 1.41 ^c^	20.90 ± 1.15	19.70 ± 1.77
OOPs-VL	19.16 ± 1.77	19.23 ± 2.51	19.48 ± 1.63	19.06 ± 1.72 ^a^	19.44 ± 1.15	18.40 ± 1.77
OOPs-L	18.99 ± 3.40	19.62 ± 1.43	17.59 ± 2.68 ^ac^	19.39 ± 2.32 ^a^	20.00 ± 0.57	19.42 ± 2.59
OOPs-M	18.43 ± 4.06	19.17 ± 1.03	18.38 ± 1.42	18.44 ± 1.90 ^a,b^	19.72 ± 1.14	19.65 ± 1.98
OOPs-H	17.86 ± 2.98	17.60 ± 1.19	17.19 ± 2.21 ^a,b,c^	18.57 ± 1.10 ^a,b^	18.05 ± 3.32 ^a^	16.79 ± 3.93 ^a,b^
WPC	18.15 ± 2.26	18.04 ± 2.29	19.03 ± 1.51	20.25 ± 1.98	20.25 ± 2.87	20.58 ± 0.93
MPC	19.09 ± 4.56	18.72 ± 1.03	19.53 ± 1.10	18.91 ± 1.46 ^a^	20.55 ± 1.34	18.28 ± 2.91
NC	4.81 ± 0.32	5.87 ± 0.19	6.12 ± 0.45	4.85 ± 0.57	5.24 ± 0.46	6.08 ± 0.33

Values are expressed as the means ± SD of 8 rats in each group. ^a^ *p* < 0.05 versus MC group, ^b^ *p* < 0.05 versus WPC group, ^c^ *p* < 0.05 versus MPC group.

**Table 3:** Effect of OOPs treatment on the OGTT-AUC.

Group	OGTT-AUC (mmol/L)	
	6th Week	10th Week
MC	55.45 ± 3.50 ^b^	48.48 ± 3.79
OOPs-VL	55.49 ± 4.27 ^b^	46.48 ± 4.91
OOPs-L	54.78 ± 4.06 ^b^	47.55 ± 3.01
OOPs-M	54.94 ± 5.27 ^b^	44.57 ± 5.52 ^b^
OOPs-H	51.84 ± 2.44 ^a^	43.23 ± 4.46 ^a,b,c^
WPC	49.73 ± 3.25 ^a,c^	49.56 ± 4.29
MPC	55.45 ± 2.06 ^b^	47.66 ± 3.44
NC	12.87 ± 1.22 ^a,b,c^	13.03 ± 1.37 ^a,b,c^

Values are expressed as the means ± SD of 8 rats in each group. ^a^ *p* < 0.05 versus MC group, ^b^ *p* < 0.05 versus WPC group, ^c^ *p* < 0.05 versus MPC group.

**Table 4:** Effect of OOPs treatment on the OGTT-2nd hour glucose.

Group	OGTT-2nd Hour Glucose	
	6th Week	10th Week
MC	24.70 ± 2.90	22.33 ± 1.81
OOPs-VL	24.13 ± 2.30	20.99 ± 2.27
OOPs-L	22.97 ± 1.33	21.17 ± 2.15
OOPs-M	24.16 ± 2.00	20.15 ± 2.81
OOPs-H	22.76 ± 1.89 ^a^	19.77 ± 2.71 ^a,b^
WPC	23.38 ± 1.88	22.22 ± 2.02
MPC	22.58 ± 1.74	21.62 ± 2.02
NC	5.56 ± 0.79 ^a,b,c^	5.49 ± 0.52 ^a,b,c^

Values are expressed as the means ± SD of 8 rats in each group. ^a^ *p* < 0.05 versus MC group, ^b^ *p* < 0.05 versus WPC group, ^c^ *p* < 0.05 versus MPC group.

**Table 5:** Effect of OOPs treatment on the SOD and MDA.

Group	SOD(U/L)	MDA (nmol/L)
MC	155.88 ± 18.58	4.43 ± 0.60
OOPs-VL	173.43 ± 21.00	3.84 ± 0.72
OOPs-L	162.03 ± 29.76	3.89 ± 0.69
OOPs-M	156.31 ± 19.31	4.10 ± 0.66
OOPs-H	177.48 ± 33.71 ^a^	4.24 ± 0.48
WPC	173.50 ± 21.20	4.07 ± 0.70
MPC	161.10 ± 26.10	3.97 ± 0.69
NC	170.19 ± 29.05	4.15 ± 0.65

Values are expressed as the means ± SD of eight rats in each group. ^a^ *p* < 0.05 versus MC group.

**Table 6:** Effect of OOPs treatment on the ALT and AST.

Group	ALT(U/L)	AST(U/L)
MC	200.83 ± 49.56	244.17 ± 83.95
OOPs-VL	193.16 ± 72.24	217.20 ± 68.13
OOPs-L	169.00 ± 37.15	216.17 ± 62.14
OOPs-M	183.33 ± 45.29	216.57 ± 57.45
OOPs-H	133.13 ± 49.39	200.25 ± 68.18
WPC	172.50 ± 46.71	251.83 ± 60.29
MPC	265.00 ± 19.97	319.33 ± 7.02
NC	68.38 ± 16.86 ^a^	139.00 ± 18.97 ^a^

Values are expressed as the means ± SD of eight rats in each group. ^a^ *p* < 0.05 versus MC group.

**Table 7:** Effect of OOPs treatment on the micturition frequency and urine volume.

Group	Micturition Frequency	Urine Volume (mL)
MC	25.40 ± 9.53	78.90 ± 14.34
OOPs-VL	25.05 ± 10.10	80.23 ± 18.61
OOPs-L	20.20 ± 11.34	61.93 ± 22.74
OOPs-M	20.20 ± 7.36	56.15 ± 26.11
OOPs-H	18.80 ± 9.04	48.03 ± 15.66 ^a^
MPC	22.40 ± 8.68	34.48 ± 20.01 ^a^
NC	13.60 ± 7.23 ^a^	16.50 ± 8.70 ^a^

Values are expressed as the means ± SD of 6 rats in each group. ^a^ *p* < 0.05 versus MC group.

**Table 8:** Amino acid composition of oat oligopeptides.

Amino Acid	Amino Acid Composition OOPs (g/100g)
Glu	13.61
Asp	5.62
Leu	4.66
Ala	4.43
Gly	4.24
Arg	4.08
Val	3.72
Pro	3.67
Ser	3.45
Phe	3.30
Lys	2.82
Thr	2.73
Tyr	2.72
Ile	2.47
Cys	1.89
His	1.46
Met	1.26
Total	66.13
